# Study of Water Adsorption on EDTA-Modified LTA Zeolites

**DOI:** 10.3390/nano12081352

**Published:** 2022-04-14

**Authors:** Janez Volavšek, Oleksii Pliekhov, Olena Pliekhova, Gregor Mali, Nataša Zabukovec Logar

**Affiliations:** 1 National Institute of Chemistry, Hajdrihova 19, 1000 Ljubljana, Slovenia; janez.volavsek@ki.si (J.V.); oleksii.pliekhov@eternit.si (O.P.); gregor.mali@ki.si (G.M.); 2 Faculty of Mathematics and Physics, University of Ljubljana, Jadranska 19, 1000 Ljubljana, Slovenia; 3 School of Science, University of Nova Gorica, Vipavska 13, 5000 Nova Gorica, Slovenia; olena.pliekhova@eternit.si

**Keywords:** 4A zeolite, 5A zeolite, LTA topology, water adsorption, EDTA dealumination, ^27^Al NMR, ^29^Si NMR

## Abstract

The present work deals with the study of water adsorption on acid-modified zeolites A. Commercial zeolites 4A (Na form) and 5A (Ca form) were subjected to EDTA dealumination, and their structure, textural properties and stability were checked by XRD, EDX, NMR and N_2_ physisorption analyses. The water adsorption isotherms of the parent zeolites and their modified forms were measured at a temperature of 25 °C and up to a relative pressure of 0.9. The results show that the treatment with EDTA drastically changes the structural properties of the zeolites and increases the water adsorption capacity by up to 10%. The changes depend on the type of extra-framework cations (Na^+^ and Ca^2+^) and the EDTA concentration.

## 1. Introduction

Adsorption of water on zeolites is an important process, applicable for multiple industrial purposes. For instance, in natural gas separation, water vapor is the most undesirable impurity, which can be effectively removed using zeolite molecular sieves [1]. Moreover, in post-combustion CO_2_ capture, it has been shown that when the water content in the feedstock is high, the use of a water-absorbing zeolite pre-layer is essential to prevent failure of the carbon capture process [2]. On the other hand, in heterogeneous catalysis, the adsorption of water molecules in the pores of zeolite catalysts affects the selectivity and the rate of the catalytic reaction [3]. Recently, studies on water adsorption in zeolites have been further promoted due to the potential of zeolites as sorption-based heat storage and reallocation materials with water as the working fluid [4]. The two-step process involves endothermic desorption of water with available solar or waste heat to obtain activated zeolite and exothermic adsorption of water in the material when the heat is needed. The two critical parameters that determine the usability and storage performance of the selected zeolite are the water adsorption capacity and the water desorption temperature, which determine the temperature of the heat that can be stored.

Zeolites are crystalline, porous aluminosilicates in which the negative charge of the framework is balanced by extra-framework cations, such as sodium or calcium. The interactions of water with zeolites and the amount of adsorbed water for a particular application can be influenced by optimizing the chemical composition, pore size and particle size distribution [5]. Zeolites with low silica content and a Si/Al ratio approaching one generally exhibit more hydrophilic characteristics than high silica zeolites with a high Si/Al ratio. In addition, the hydrophilicity of zeolites can be influenced by the nature of the charge-balancing cations in the pores. For example, the larger electrostatic charge of divalent calcium cations compared to monovalent sodium cations leads to a stronger interaction with the dipole of water [6]. One of the most important members of the zeolites with great performance in adsorption applications is zeolite A with LTA topology and a Si/Al ratio of 1 [7]. Each unit cell contains a supercage (α-cage) with a diameter of 11.4 Å and eight smaller cages (β-cage) with a diameter of 6.6 Å. The LTA zeolite exhibits strong preferential adsorption of water because of its small pore openings to the cages, which hinder the diffusion of some larger molecules, as well as the polar nature of the zeolite framework due to the low Si/Al ratio. The exchange of sodium charge-balancing cations for larger potassium ones reduces the effective pore openings from ~4 to ~3 Å and consequently its water adsorption capacity. On the other hand, the calcium-exchanged LTA zeolite possesses larger pore openings with a diameter of ~5 Å, which enhances the maximum water loading, as well as the hydrophilicity of the material.

The pore sizes can be further increased by reducing the aluminum concentration in the framework, i.e., by dealumination, which is associated with a lower amount of charge-balancing extra-framework cations and also with altered interactions of water with the framework [8]. For example, our recent investigation of water adsorption in zeolite Y for heat storage and reallocation applications has shown that increasing the Si/Al ratio of the framework significantly lowers the desorption temperature and affects the water sorption capacity [9]. Generally, the Si/Al ratio of silica-rich zeolites (zeolite Y, mordenite) may be significantly increased by using dealuminating methods such as acid treatment or steaming [8]. Such treatments cause almost no serious damage to the zeolite framework due to the already high Si/Al ratio. In contrast, dealumination of the LTA framework type zeolites is a very sensitive process due to the high Al content and low Si-to-Al ratio. Thus, methods such as steaming or acid leaching cannot be applied. Dealumination of LTA zeolites can only be achieved under carefully controlled, mild reaction conditions using ammonium fluorosilicate as a dealumination agent [10,11,12,13]. By using this technique, the authors were able to produce mesoporous materials with a rather narrow pore size distribution and significantly changed textural and sorption properties. Ammonium fluorosilicate, however, according to the globally harmonized system of classification and labeling of chemicals (GHS), belongs to the dangerous class of substances. Ethylenediaminetetraacetic acid (EDTA), on the other hand, is water-soluble nontoxic aminopolycarboxylic acid, which finds its wide application in industry because of its strong ability to sequester metal ions such as Al^3+^, Fe^3+^, Ca^2+^, etc. Moreover, it has been postulated that EDTA can be used for dealumination of zeolite Y under mild reaction conditions, also by our group [8,9,14]. However, there is to our knowledge no information available about the application of EDTA for dealumination of LTA-type zeolites.

The presented research was thus focused on the study of the effects of EDTA dealumination of two forms of LTA zeolites. The aim was to determine the structural and textural changes of sodium LTA zeolite 4A and Ca-exchanged form 5A upon dealumination, which has not been studied up to date. Furthermore, we evaluated the water adsorption properties of dealuminated LTA materials for possible use in adsorption applications, with an emphasis on heat storage and reallocation, which included determination of water sorption capacities, as well as temperature and heat of desorption.

## 2. Materials and Methods

### 2.1. Materials

The parent 4A and 5A zeolites were commercially available materials supplied by Silkem Company, Kidričevo, Slovenia. Dealumination with the acid form of EDTA started with 5 g of the initial as-obtained zeolite, which was refluxed for 6 h with 150 mL of EDTA solution of the selected concentration. After the treatment, the particles within the suspension were filtered off and washed with distilled water until neutral pH was reached. Finally, the samples were air-dried at 60 °C. The obtained dealuminated samples were denoted as Z4A-x and Z5A-x, where x denotes the concentration of EDTA in mmol/L (x = 8, 17, 34).

### 2.2. Methods

#### 2.2.1. X-ray Powder Diffraction (XRD)

Powder X-ray diffractograms were recorded on a PANalytical X’Pert PRO diffractometer (Malvern Panalytical, Almelo, The Netherlands), using Cu Kα radiation with λ = 1.5406 Å. The diffractograms were recorded in the 2θ range between 5 and 50° with an acquisition time of 100 s per 0.034° step. Data were collected with a fully opened X’Celerator detector (Malvern Panalytical, Almelo, The Netherlands). We estimated the degree of crystallinity by comparing the area of all of the diffraction peaks of a given sample (S_X_) in the 2θ range between 20 and 30° to the area of the same peaks of the reference zeolite (S_R_) [9,15]. Thus

Percent crystallinity of zeolite = S_X_/S_R_ × 100 (1)

#### 2.2.2. Scanning Electron Microscopy (SEM)

Microstructural analysis was carried out using a Zeiss Supra™ 3VP scanning electron microscope equipped with energy dispersive X-ray (EDX) detectors (Carl Zeiss Microscopy, Jena, Germany).

#### 2.2.3. Solid-State Nuclear Magnetic Resonance (NMR)

Solid-state NMR spectra were recorded on a 600 MHz Varian spectrometer, using a 1.6 mm HXY CPMAS probe. Larmor frequencies were 156.20 MHz and 119.09 MHz for ^27^Al and ^29^Si nuclei, respectively. All samples were spun at 20 kHz. For ^27^Al magic-angle spinning (MAS) NMR measurements, a 1.0 µs excitation pulse was used; 1600 scans were collected, and delay between the scans was 0.5 s. In ^29^Si MAS NMR measurements, a 90-degree excitation pulse of 1.6 µs and repetition delay of 30 s were used; the number of repetitions was 2000. The deconvolution of spectra and corresponding relative integrals were produced with DMFit software (Release No.: 20200306), which can be found on the following website: https://nmr.cemhti.cnrs-orleans.fr/dmfit/ (accessed on 14 May 2020) [16]. The precision of simulation of NMR spectra, for the purpose of deconvolution of experimental spectra, was evaluated by a method of tripling the values of the standard deviations on reported parameters (peak shifts–positions and their relative integrals–areas under the curve). These were obtained from 300 steps with the DMFit Monte Carlo errors model subroutine (Supplementary Material, Table S1); the errors were omitted in the main text for clarity.

#### 2.2.4. Nitrogen Adsorption Measurements

N_2_ physisorption measurements were carried out on a Tristar 3000 volumetric adsorption device (Micromeritics, Norcross, GA, USA) at 77 K. The BET-specific surface area (S_BET_) was obtained from the adsorption data in a relative pressure range between 0.01 and 0.1. The total pore volume (V_TOT_) was calculated from the amount of nitrogen adsorbed at a relative pressure of 0.95. The external surface area (S_EXT_) and micropore volume (V_MIC_) were determined using the t-plot method in the range of relative pressures between 0.05 and 0.35. The mesopore volume (V_MES_) was obtained as the difference between the total pore volume and the micropore volume.

#### 2.2.5. Thermogravimetric Analysis (TGA)

Thermogravimetric analysis (TGA) was performed on a Q5000IR (TA Instruments, Inc., New Castle, DE, USA) analyzer. The air flow was 25 mL min^−1^, the temperature range was 25 to 700 °C and heating rate was 10 °C min^−1^. Before the TG measurements were performed, the samples were exposed to an atmosphere with room temperature and relative humidity of 75% (provided by the saturated solution of ammonium chloride). After 3 days in this atmosphere, fully hydrated samples were obtained.

#### 2.2.6. Differential Scanning Calorimetry (DSC)

Dynamic calorimetric measurements were carried out on a Q2000 DSC instrument (TA Instruments, Inc., New Castle, DE, USA) in the temperature range between 25 and 400 °C. The heating ramp was 1 °C min^−1^.

#### 2.2.7. Fourier-Transform Infrared Spectroscopy (FTIR)

For the IR measurements, the samples were dispersed in KBr pellets. The spectra were obtained on a FTIR Spectrum 100 spectrometer (Perkin Elmer, Inc., Shelton, CT, USA) with a 4 cm^−1^ resolution in the range of 4000–400 cm^−1^.

#### 2.2.8. Water Vapor Adsorption Measurements

We measured the water vapor adsorption isotherms on a IGA-100 gravimetric analyzer (Hiden Isochema Ltd., Warrington, UK). The samples were first activated at 300 °C under high vacuum for 3 h. The isotherms were measured at 25 °C up to a relative pressure of 0.9. Equilibrium time was 80 min for all the measurements.

## 3. Results and Discussion

### 3.1. Materials Characterization

#### 3.1.1. XRD Analysis

**Figure 1:**
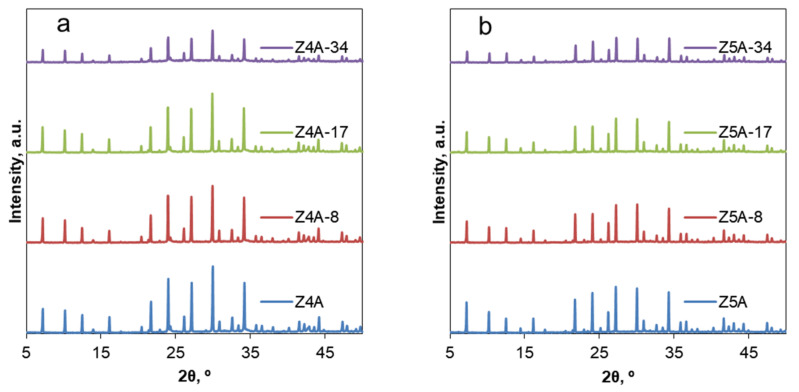
XRD patterns of the parent and EDTA dealuminated zeolite 4A (**a**) and zeolite 5A (**b**) samples.

X-ray diffraction analysis showed that the commercial aluminosilicates were highly crystalline and exhibited the LTA topology (Figure 1a,b). The XRD patterns of modified zeolite 4A (Figure 1a) and zeolite 5A (Figure 1b) samples obtained with different concentrations of EDTA show changes in diffraction peak intensities. With the increase in the EDTA concentration under the same reaction conditions, the relative crystallinity gradually decreases (Table 1). This indicates that the crystalline structure was partially damaged. It can be concluded that higher concentrations of EDTA caused lower crystallinity, reaching the lowest level of 45% and 58% of relative crystallinity for Z4A-34 and Z5A-34 samples, respectively.

#### 3.1.2. EDX Elemental Analysis

EDX analysis of the parent samples revealed the following chemical composition: Na_0.12_Al_0.12_Si_0.12_O_0.48_ for zeolite 4A and Na_0.03_Ca_0.04_Al_0.12_Si_0.12_O_0.48_ for zeolite 5A. It can be seen that in the parent zeolite 5A, a significant amount of Na atoms is still present.

Results in Table 1 show that the treatment of the parent zeolite 4A with a low concentration of EDTA leads only to the removal of sodium ions, with no visible signs of dealumination. Al could be removed from the structure of zeolite 4A only by using high concentrations of EDTA. Dealumination is accompanied by a high decrease in crystallinity. In contrast, elemental analysis of the Z5A-x samples showed that EDTA treatment leads to the simultaneous removal of Al and Na, indicating the occurrence of a dealumination reaction involving both Al and Na atoms, as is in the case of EDTA dealumination of zeolite Y [17]. Along with the gradual removal of Al, EDTA treatment drastically favors the removal of remaining Na ions, leaving strongly bound Ca preserved inside the zeolite matrix. Ca ions are removed only at a higher concentration of EDTA when amorphization of the zeolite is more pronounced. EDTA treatment of zeolite 5A caused a fourfold decrease in the overall amount of Na, lowering it from 3 atomic percent in the parent Z5A sample to 0.8 atomic percent in the Z5A-34 sample. The amount of Na ions after EDTA treatment of zeolite 4A decreased from 12 atomic percent in the parent Z4A sample to 7.6 atomic percent in Z4A-34.

#### 3.1.3. FTIR Spectra Analysis

**Figure 2:**
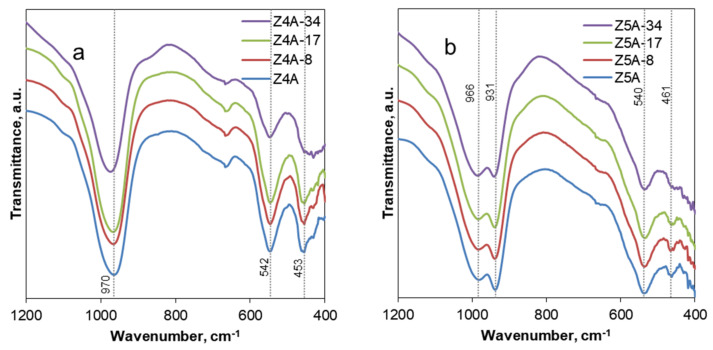
FTIR spectra of the parent and EDTA dealuminated zeolite 4A (**a**) and zeolite 5A (**b**) samples.

The removal of Al atoms from the zeolite framework was confirmed with FTIR analysis. Figure 2 presents the IR spectra of the zeolite 4A series of samples (Figure 2a) and 5A series of samples (Figure 2b) with characteristic peaks associated with the SiO_4_ and AlO_4_ framework tetrahedra at the regions of 800–1200 cm^−1^ and 400–600 cm^−1^ [18].

The results show a high-frequency shift of the bands associated with the asymmetric stretching vibrations of bridge bonds Si-O-Si(Al) at 800–1200 cm^−1^ due to the decrease in aluminum content. In the case of Z4A samples, a frequency shift can be detected only for the Z4A-34 sample where a high concentration of EDTA was used, which is in agreement with the results of the chemical analysis. Furthermore, there is a decrease in peak intensity at 400–500 cm^−1^. This peak corresponds to the vibrations of the O-Si(Al)-O bridge bonds [19], so the decrease in its intensity points to a partial collapse of the structure.

#### 3.1.4. Solid-State NMR Analysis

**Figure 3:**
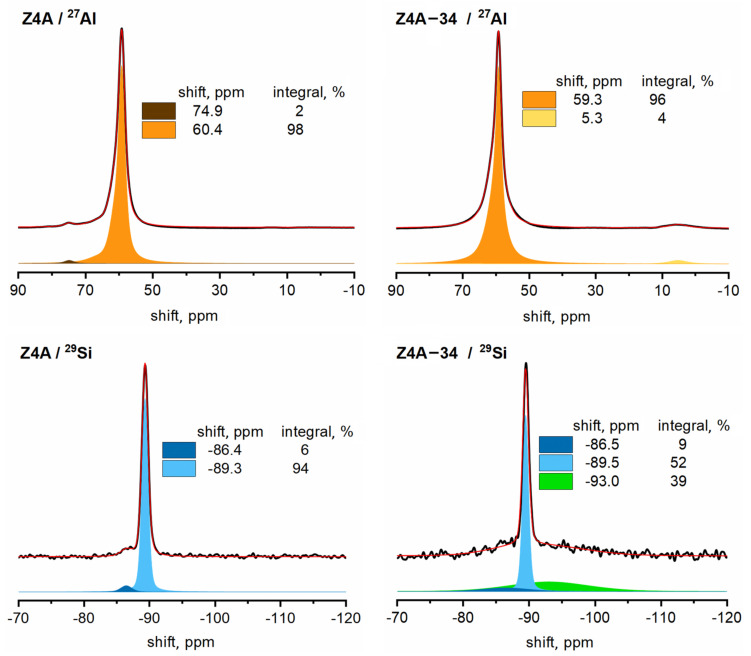
^27^Al and ^29^Si MAS NMR spectra of Z4A and Z4A-34 (black lines = measured spectra, red lines = modeled spectra, color filled areas under the curve = individual contributions to the modeled spectra).

Z4A, Z4A-34, Z5A and Z5A-34 samples were also inspected by solid-state NMR spectroscopy. The ^27^Al and ^29^Si MAS NMR spectra of Z4A (Figure 3) both exhibit one strong, sharp signal, typical for Na-A zeolites. The Al signal at about 60 ppm belongs to tetrahedrally coordinated framework aluminum and the Si signal at −89.3 ppm to the Si(4Al) species [20], which are expected in a zeolite with a Si/Al ratio of 1 and in which the Si and Al atoms are strictly alternating on the framework tetrahedral sites. Both ^27^Al and ^29^Si MAS NMR spectra also exhibit one very weak signal, which might belong to very rare defects (e.g., to Al(3Si,OH) and Si(3Al,OH) species). The ^27^Al MAS NMR spectrum of Z4A-34 is somewhat different from the spectrum of the parent material. The main signal of the tetrahedral Al is slightly shifted and broadened, and a new weak signal at about 5 ppm can be detected. The latter signal most probably belongs to extra-framework Al(OH)_3_ or similar species [21].

If EDTA treatment of Z4A does not significantly affect the silicon content in the sample, it is reasonable to normalize the ^29^Si MAS NMR spectra of Z4A and Z4A-34 so that their integrals are equal. After such normalization, the ^27^Al MAS NMR spectra offer a solid quantitative comparison of the total amount of Al within the samples. The comparison shows that Z4A-34 contains about 20% less aluminum than Z4A, which agrees rather well with the results of the elemental analysis. The ^29^Si MAS NMR spectrum exhibits three contributions; in addition to the main, narrow signal at −89.5 ppm, we can also detect a weak, rather broad signal at −86.5 ppm and a very broad and quite strong signal at −93 ppm. This latter signal contributes almost 40% to the total silicon signal. Both broad signals become significantly enhanced in the ^1^H-^29^Si CPMAS experiment (Supplementary Material, Figure S1), suggesting that they both belong to silicon atoms with at least one OH group attached. Since the change in environment from Si(4Al) to Si(3Al,OH) should shift the corresponding Si NMR signal from about −90 ppm towards −80 ppm [22], the signal at −93 ppm should be ascribed to silicon species that not only comprise OH group(s) in their coordination sphere, but also silicon atoms, i.e., this broad signal should be ascribed to Si(2Al,1Si,OH), Si(1Al,2Si,OH) and perhaps even Si(2Si,2OH) species [23]. The NMR analysis of Z4A-34 thus not only shows that treatment of Z4A with EDTA removes aluminum from the framework of the zeolite Z4A, but also suggests that the obtained sample is actually a mixture of two types of materials. About 50–60% of the silicon is within an intact zeolite Na-A, and about 40–50% of silicon belongs to a disordered phase with a significantly higher Si/Al ratio and a significant fraction of defects, which can probably be found at the increased surface.

**Figure 4:**
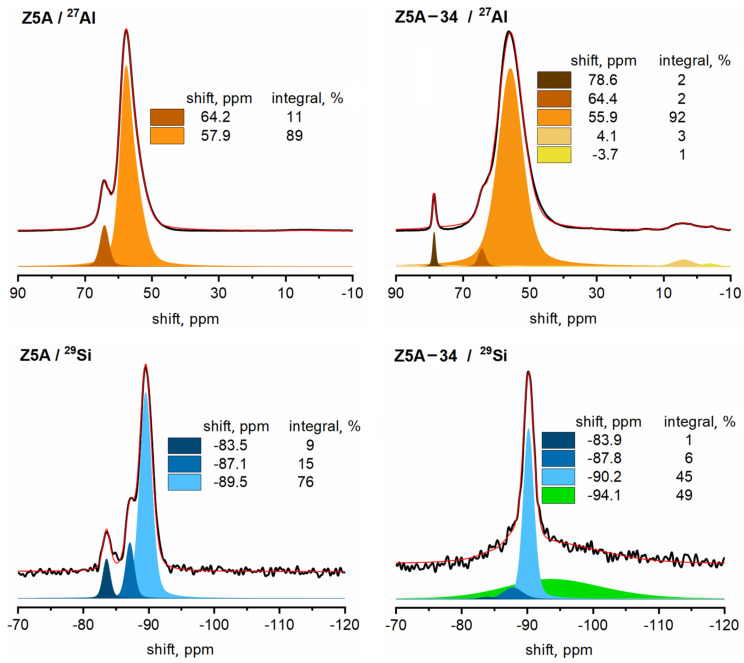
^27^Al and ^29^Si MAS NMR spectra of Z5A and Z5A-34 (black lines = measured spectra, red lines = modeled spectra, color filled areas under the curve = individual contributions to the modeled spectra).

The ^27^Al and ^29^Si MAS NMR spectra of Z5A are notably different from the corresponding spectra of Z4A (Figure 4). The main Al signal that can be assigned to framework tetrahedral Al [20] is broader and shifted to 58 ppm, and there is also a non-negligible well-resolved signal at 64 ppm, the origin of which is not entirely clear. Perhaps it belongs to framework aluminum affected by Ca(OH)^+^ cations [24]. The ^29^Si MAS NMR spectrum exhibits three signals: the strongest resonates at −89.5 ppm, a weaker one at −87.1 ppm and the weakest at −83.5 ppm. The latter signal becomes strongly enhanced in the ^1^H-^29^Si CPMAS experiment, so it could be assigned to Si(3Al,OH) species (Figure S1). The signal at −87.1 ppm is less enhanced by the polarization transfer from protons, suggesting that it is more distant from the hydrogen atoms. Perhaps this signal belongs to Si atoms related to the above-discussed Al atoms, which could potentially be (both) affected by Ca(OH)^+^ cations. The ^27^Al and ^29^Si MAS NMR spectra of the EDTA-treated Z5A-34 are significantly changed. The strongest Al signal is still broader and further shifted to 56 ppm. Three new signals also appear; the two resonating at about 4 ppm and −4 ppm belong to extra-framework octahedral aluminum, whereas the sharp signal at 79 ppm can be assigned to extra-framework four-coordinated Al(OH)_4_^−^ aluminate ions [25]. Quantitative comparison of the aluminum NMR spectra of Z5A and Z5A-34 shows that, again, about 20% of aluminum was removed during EDTA dealumination. The ^29^Si MAS NMR spectrum of Z5A-34 is qualitatively similar to the corresponding spectrum of Z4A-34. In addition to the signals resonating at −90, −87.8 and −83.9 ppm, it exhibits a very strong, very broad signal with a peak maximum at −94 ppm. This latter signal contributes about 50% to the total silicon signal and leads to similar conclusions as the broad signal detected in the spectrum of Z4A-34, i.e., this broad signal should be ascribed to Si(2Al,1Si,OH), Si(1Al,2Si,OH) or even Si(2Si,2OH) species, which is in accordance with removal of Al from the framework [23]. This confirms that EDTA treatment of Z5A leads to partial dealumination, partial destruction of crystallinity and to an increase in the surface. In our previous study of EDTA-treated zeolite Y with Si/Al ratios from 2.5 to 11.2, the NMR analysis showed that the contribution of OH groups on the framework was negligible. Moreover, a signal for pure silicate domains, which contributed to around 12% of the total silicon content, was detected [14]. These observations confirm that dealumination of high Si/Al zeolites is more challenging and tends to favor hydrolysis reactions and associated degradation of crystalline structure [26].

### 3.2. Textural Characterization

#### 3.2.1. Nitrogen Adsorption Study

The textural properties of the parent and EDTA dealuminated zeolite 4A and 5A are presented in Table 2.

**Figure 5:**
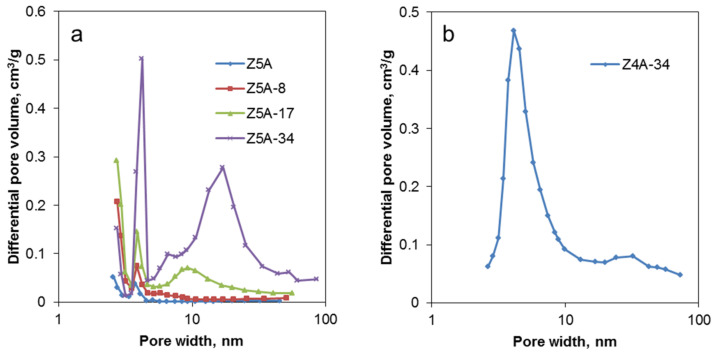
Pore size distribution of parent and EDTA dealuminated zeolite 5A (**a**) and Z4A-34 sample (**b**). For Z4A, Z4A-8 and Z4A-17, no evaluable measurements could be obtained, in accordance with the literature data on Na^+^ cations blocking the entrance to the *α*-cages for nitrogen molecules [27].

The results show that chemical treatment with EDTA drastically changes the textural properties of the Z5A samples. Experimental data on N_2_ physisorption reveal that an increase in EDTA concentration leads to a decrease in specific BET surface area and, at the same time, to a larger total pore volume and external surface area, indicating the introduction of secondary mesopores into the samples. The latter is accompanied by the reduction of the micropore volume. Figure 5a shows the distribution of pore sizes in Z5A-x samples. Modification of the parent zeolite 5A with a low concentration of EDTA leads to an increase in the amount of mesopores 4 nm in diameter. Such mesopores are also present in the parent material in a very low amount. This effect is probably related to the leaching of sodium ions out of the zeolite structure. At higher EDTA concentrations, mesopores with diameters in the range of 5 to 30 nm start to appear.

It is difficult to follow the modifications of zeolite 4A by N_2_ physisorption, as no significant adsorption of nitrogen was detected for Z4A, Z4A-8 and Z4A-17 samples (Supplementary Materials, Figure S2, Table S2). Treatment with higher EDTA concentration in the Z4A-34 sample leads to the formation of a mesoporous material with a fairly homogenous system of mesopores and an average pore diameter of about 4 nm (Figure 5b). The absence of N_2_ adsorption at the parent zeolite 4A, and, in our case, at the modified Z4A-8 and Z4A-17 samples, was previously observed in [27]. This could be explained as a result of limited diffusion of relatively bulky N_2_ molecules, which have a kinetic diameter of 3.64 Å, into the pores of sodium form of zeolite A with pore openings of about 4 Å in diameter and location of Na^+^ cations in zeolite 4A at the entrance to the *α*-cages. This issue could be eliminated with the adsorption of smaller molecules, such as water, which has a kinetic diameter of 2.65 Å.

#### 3.2.2. Water Vapor Adsorption Isotherms

The specific surface area obtained from the water adsorption measurements on the parent Z4A sample was 398 m^2^/g and increased gradually with the increase in EDTA concentration to 541 and 571 m^2^/g for Z4A-8 and Z4A-17 samples, respectively. These results are in good agreement with the elemental analysis data; thus, we can conclude that the removal of sodium atoms provides more space for adsorbed water molecules and gives rise to a higher surface area. However, a further increase in EDTA concentration in the Z4A-34 sample resulted in a decreased surface area to the value of 440 m^2^/g, which could be explained by the partial amorphization of the sample. The same trend is observed for Z5A-x samples. The specific surface area obtained from water adsorption data for the parent Z5A sample was 543 m^2^/g, which was increased after the course of chemical treatment with EDTA to 617 and 630 m^2^/g for Z5A-8 and Z5A-17 samples, respectively. A further increase in EDTA concentration in the Z5A-34 sample resulted in a decrease in surface area to the value of 562 m^2^/g.

**Figure 6:**
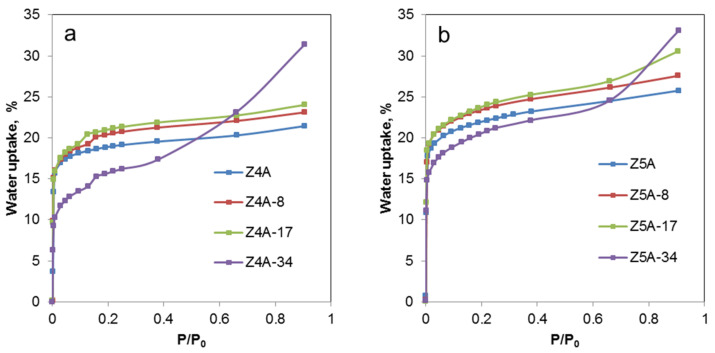
Water adsorption isotherms of parent and EDTA dealuminated zeolite 4A (**a**) and 5A samples (**b**).

Figure 6 shows the water adsorption isotherms for the parent and EDTA-treated samples of Z4A and Z5A. The shape of the adsorption isotherm of the parent zeolites shows typical hydrophilic characteristics with quick saturation at low partial pressures. Treatment with EDTA slightly changes the shape of the isotherms, probably due to the decrease in relative crystallinity and the increasing contribution of the amorphous phase. The samples treated with EDTA exhibit a higher water uptake than the initial samples. In the case of zeolite 4A with 21 weight percent of initial capacity, modification with EDTA contributed to an increase to 23, 25 and 31% for Z4A-8, Z4A-17 and Z4A-34, respectively. Similarly, for zeolite 5A the initial capacity of 26 weight percent increased to 28, 31 and 34% for Z5A-8, Z5A-17 and Z5A-34, respectively. The observed increase in overall water uptake can be associated with the formation of a secondary mesoporous system in the EDTA-treated samples.

### 3.3. Thermal Characterization

The thermal characteristics of the samples were assessed by using thermogravimetric and calorimetric measurements. The results of the analysis are summarized in Table 3.

As can be seen, the maximum temperature of desorption and integral heat of desorption change with the increase in EDTA concentration. The higher affinity to water molecules is likely attributed to the increased leaching of Na ions and negligible removal of Al when lower concentrations of EDTA (8–17 mmol/L) were used. Both parameters drop significantly at a high concentration of EDTA for both zeolites 4A and 5A when the removal of Al atoms from the zeolite crystals became more pronounced. The reason for this observation is the reduced electrostatic field in the pores of the zeolites caused by the lower Al content in the zeolite framework [28].

## 4. Conclusions

In this work, we demonstrated that treatment of 4A and 5A zeolites with non-toxic ethylenediaminetetraacetic acid resulted in the production of microporous/mesoporous materials with narrow pore size distributions. The nature of the extra-framework cations had little effect on the changes in textural properties of the EDTA-treated samples. In both cases, total pore volume and external surface area increased with treatment at lower EDTA concentrations (8 and 17 mmol/L EDTA) and then decreased at the highest concentration of 34 mmol/L EDTA. Zeolite 5A exhibited up to 25% higher values at all concentrations, consistent with the size and distribution of sodium and calcium atoms in the zeolite structure.

Elemental composition and FTIR analysis revealed that the treatment resulted in dealumination of the framework by removing up to one-fifth of the Al and reducing the amount of extra-framework cations at the highest EDTA concentration. At lower concentrations, mostly extra-framework cations were removed from both zeolites, and more protonic LTA zeolites were formed, which are kinetically less stable than cationic zeolites and more susceptible to framework hydrolysis and dealumination [26].

XRD showed that the crystallinity of the structures decreased with increasing EDTA concentration to half of the original crystallinity. Moreover, solid-state NMR analysis confirmed the decrease in Al concentration in the framework while showing that the modification caused a considerable number of defects in the structure. This was evident from the presence of a considerable amount of silanol OH groups on the framework. NMR analysis showed that about half of the structure in both dealuminated LTA zeolites remained intact in terms of Si-O-Al connectivity, while the other half, with a higher Si/Al ratio, was disordered. This is related to the inability of EDTA to penetrate deeply into the LTA zeolite crystal.

The evaluation of water sorption performance showed that the obtained materials with modified porosity showed improved water adsorption properties compared to unmodified samples. Regarding the sorption capacity for water, the initial capacity for unmodified zeolites was 21% and 26% for 4A and 5A, respectively. EDTA treatment resulted in an increase in capacity, i.e., 31% and 34% for 4A and 5A, respectively. The changes in desorption temperature and heat of adsorption during EDTA treatment are more complex. Initially, at low EDTA concentrations, both values increased for both zeolites. This can be related to the migration of cations during dehydration to the entrances of the pores, which hinders water diffusion from the pores [29]. At the highest EDTA concentration, where the amount of mesoporosity is considerable and, on the other hand, the amount of aluminum and extra-framework cations is the lowest, the diffusion limitations and the interactions between water and zeolite are the lowest, the desorption temperature and the heat of adsorption decreased close to or below the values of the untreated samples.

We can conclude that EDTA treatment has proven successful in modifying the structural and textural properties of LTA zeolites. Improved water sorption capacities and low water desorption temperatures make the modified materials promising materials for heat storage applications as well, but further studies on hydrothermal stability under multiple cycles of water adsorption and desorption are needed.

## Figures and Tables

**Table 1:** Relative crystallinity, Si-to-Al ratio and fraction of removed Na, Al and Ca cations (determined using EDX elemental analysis) from the parent zeolites 4A and 5A.

Name-C(EDTA), mmol/L	Relative Crystallinity, %	Si/Al ± 0.04	Na_removed_, % ±2%	Al_removed_, % ±2%	Ca_removed_, % ±2%
Z4A	100	1.01	0	0	-
Z4A-8	81	1.03	14	0	-
Z4A-17	74	1.03	17	2	-
Z4A-34	45	1.23	39	17	-
Z5A	100	1.04	0	0	0
Z5A-8	82	1.10	36	2	0
Z5A-17	75	1.15	52	7	0
Z5A-34	58	1.28	72	19	12

**Table 2:** The textural properties of the parent and EDTA dealuminated zeolite 4A and 5A.

Name-C(EDTA), mmol/L	S_BET_, m^2^/g	S_EXT_, m^2^/g	V_TOT_, cm^3^/g	V_MIC_, cm^3^/g	V_MES_, cm^3^/g	Average Pore Diameter, nm
Z4A-34	103	103	0.19	0	0.19	7.5
Z5A	602	64	0.29	0.25	0.04	1.9
Z5A-8	561	105	0.29	0.21	0.08	2.1
Z5A-17	502	128	0.30	0.19	0.11	2.3
Z5A-34	418	142	0.35	0.13	0.22	3.4

S_BET_: BET-specific surface area; S_EXT_: external surface area; V_TOT_: total pore volume; V_MIC_: micropore volume; V_MES_: mesopore volume.

**Table 3:** Maximum desorption temperature (T_MAX_) and integral heat of desorption (Q_INT_) of the parent and EDTA dealuminated zeolite 4A and 5A.

Name-C(EDTA), mmol/L	T_MAX_, °C	Q_INT_, kJ/g
Z4A	131	0.30
Z4A-8	138	0.31
Z4A-17	142	0.33
Z4A-34	122	0.17
Z5A	173	0.58
Z5A-8	178	0.59
Z5A-17	194	0.62
Z5A-34	175	0.49

## Data Availability

The supporting data are available on the journal webpage and from the authors.

## Supplementary Materials

The following supporting information can be downloaded at: https://www.mdpi.com/article/10.3390/nano12081352/s1, Figure S1: ^29^Si MAS and ^1^H-^29^Si CPMAS NMR spectra of Z4A-34 and Z5A-34, Figure S2: N_2_ physisorption isotherms and pore size distribution of Z4A, Z4A-8 and Z4A-17; Table S1: Reported parameters with their errors for NMR spectra for Z4A, Z4A-34, Z5A and Z5A-34; Table S2: Textural data for Z4A, Z4A-8 and Z4A-17.
