# Supplementary material for: Study of Water Adsorption on EDTA-Modified LTA Zeolites
Source: Nanomaterials (Basel). 2022 Apr 14;12(8):1352. doi: 10.3390/nano12081352 (PMC9027755; doi:10.3390/nano12081352)
Supplement: Supplementary file 1 [file nanomaterials-12-01352-s001.zip › nanomaterials-1654136-supplementary.pdf]

nanomaterials

Supplementary Information

Study of Water Adsorption on EDTA-Modified LTA Zeolites

Janez Volavšek [1,2], Oleksii Pliekhov [1,†], Olena Pliekhova [3,†], Gregor Mali [1,2,3] and Nataša Zabukovec Logar [1,3,*]

[1] National Institute of Chemistry, Hajdrihova 19, 1000 Ljubljana, Slovenia; janez.volavsek@ki.si (J.V.); oleksii.pliekhov@eternit.si (O.P.); gregor.mali@ki.si (G.M.)
[2] Faculty of Mathematics and Physics, University of Ljubljana, Jadranska 19, 1000 Ljubljana, Slovenia
[3] School of Science, University of Nova Gorica, Vipavska 13, 5000 Nova Gorica, Slovenia; olena.pliekhova@eternit.si
* Correspondence: natasa.zabukovec@ki.si; Tel.: +386-1-4760-371
† Current address: Eternit Slovenija d.o.o., Anhovo 9, 5210 Deskle, Slovenia.

1. Solid-State NMR Measurements

^{1}H-^{29}Si cross-polarization MAS (CPMAS) NMR spectra of selected samples were measured using a ramped-amplitude cross-polarization scheme with duration of 5 ms and high-power proton decoupling; repetition delay was 1 s and number of scans was 12.000.

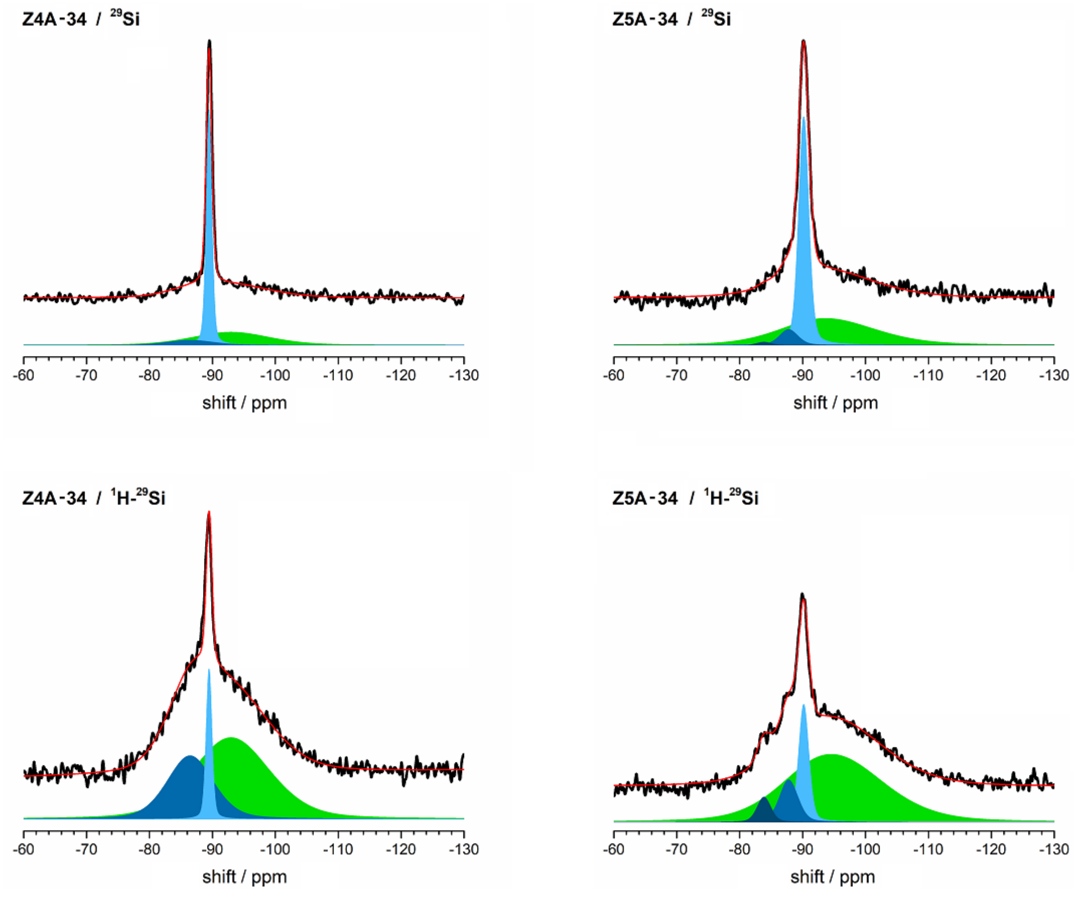

Figure S1. ^{29}Si MAS and ^{1}H-^{29}Si CPMAS NMR spectra of Z4A-34 and Z5A-34.

Table S1. Peak shifts and their relative integrals with triple values of their standard deviation obtained from the DMFit Monte Carlo errors model subroutine.

Name	Nuclei	Peak Shift, ppm	Relative Integral, %
Z4A	^{27}Al	74.9 ± 0.1	2 ± 1
		60.4 ± 0.1	98 ± 1
	^{29}Si	−86.4 ± 0.1	6 ± 1
		−89.3 ± 0.1	94 ± 1
Z4A-34	^{27}Al	59.3 ± 0.2	96 ± 6
		5.3 ± 0.1	4 ± 1
	^{29}Si	−86.5 ± 0.3	9 ± 2
		−89.5 ± 0.1	52 ± 4
		−93.0 ± 0.7	39 ± 5
Z5A	^{27}Al	64.2 ± 0.2	11 ± 1
		57.9 ± 0.1	89 ± 1
	^{29}Si	−83.5 ± 0.1	9 ± 1
		−87.1 ± 0.1	15 ± 1
		−89.5 ± 0.1	76 ± 1
Z5A-34	^{27}Al	78.6 ± 0.1	2 ± 1
		64.4 ± 0.3	2 ± 1
		55.9 ± 0.1	92 ± 2
		4.1 ± 2.1	3 ± 2
		−3.7 ± 5.6	1 ± 2
	^{29}Si	−83.9 ± 2.5	1 ± 5
		−87.8 ± 0.9	6 ± 6
		−90.2 ± 0.1	45 ± 4
		−94.1 ± 0.9	49 ± 5

2. Nitrogen Adsorption Measurements

Nitrogen physisorption measurements for Z4A, Z4A-8 and Z4A-17 were carried out at −196 °C on a Quantachrom autosorb iQ3 instrument (Haan, Germany). Before the measurement, samples were outgassed under vacuum for 12 h at 200 °C and 1 h at 300 °C. The BET specific surface area, S_{BET}, was calculated using the adsorption branch. The total pore volume, V_{TOT}, was estimated from the amount adsorbed gas at a relative pressure of 0.98. The external surface area (S_{EXT}) and micropore volume (V_{MIC}) were determined using the t-plot method at a relative pressure range from 0.05 to 0.35. The mesopore volume (V_{MES}) was calculated as the difference between the total pore volume and the micropore volume.

For Z4A again no evaluable measurement could be obtained. For Z4A-8 and Z4A-17 we were able to perform the measurement (Figure S2, Table S1), however the obtained data must be considered only informative, due to known limitations of nitrogen physisorption in Zeolite 4A evaluation. The obtained values for Z4A-8 and Z4A-17 are most probably associated with outer surface adsorption.

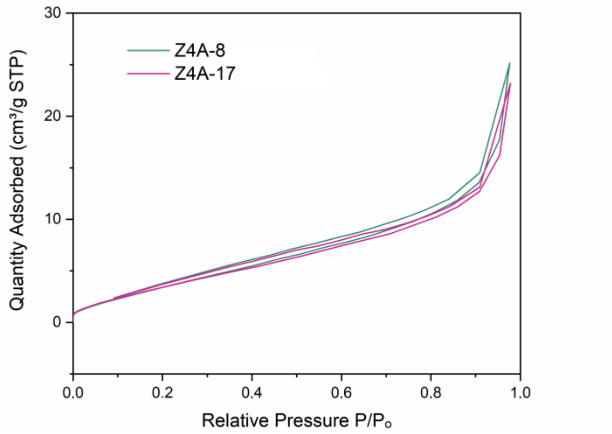

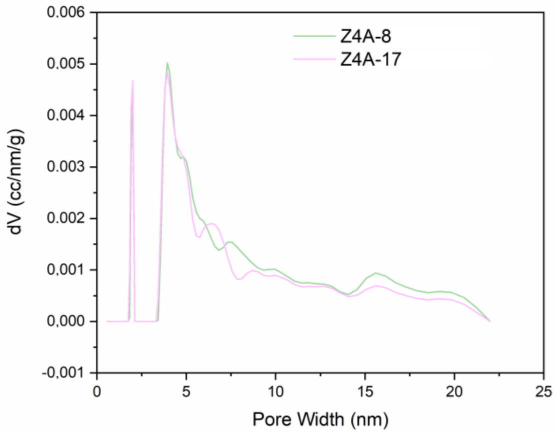

Figure S2. N_2 physisorption isotherms and pore size distribution of Z4A-8 and Z4A-17.

Table S2. Textural data for Z4A, Z4A-8 and Z4A-17.

Name-C(EDTA), mmol/L	S_{BET}, m²/g	S_{EXT}, m²/g	V_{TOT}, cm³/g	V_{MIC}, cm³/g	V_{MES}, cm³/g	Average Pore Diameter, nm
Z4A	/	/	/	/	/	/
Z4A-8	17	17	0.04	0	0.04	9.4
Z4A-17	16	16	0.04	0	0.04	9.3
